# LegumeIP 2.0—a platform for the study of gene function and genome evolution in legumes

**DOI:** 10.1093/nar/gkv1237

**Published:** 2015-11-17

**Authors:** Jun Li, Xinbin Dai, Zhaohong Zhuang, Patrick X. Zhao

**Affiliations:** Bioinformatics Lab, Plant Biology Division, Samuel Roberts Noble Foundation, 2510 Sam Noble Parkway, Ardmore, OK 73401, USA

## Abstract

The LegumeIP 2.0 database hosts large-scale genomics and transcriptomics data and provides integrative bioinformatics tools for the study of gene function and evolution in legumes. Our recent updates in LegumeIP 2.0 include gene and protein sequences, gene models and annotations, syntenic regions, protein families and phylogenetic trees for six legume species: *Medicago truncatula*, *Glycine max* (soybean), *Lotus japonicus*, *Phaseolus vulgaris* (common bean), *Cicer arietinum* (chickpea) and *Cajanus cajan* (pigeon pea) and two outgroup reference species: *Arabidopsis thaliana* and *Poplar trichocarpa*. Moreover, the LegumeIP 2.0 features the following new data resources and bioinformatics tools: (i) an integrative gene expression atlas for four model legumes that include 550 array hybridizations from *M. truncatula*, 962 gene expression profiles of *G. max*, 276 array hybridizations from *L. japonicas* and 56 RNA-Seq-based gene expression profiles for *C. arietinum*. These datasets were manually curated and hierarchically organized based on Experimental Ontology and Plant Ontology so that users can browse, search, and retrieve data for their selected experiments. (ii) New functions/analytical tools to query, mine and visualize large-scale gene sequences, annotations and transcriptome profiles. Users may select a subset of expression experiments and visualize and compare expression profiles for multiple genes. The LegumeIP 2.0 database is freely available to the public at http://plantgrn.noble.org/LegumeIP/.

## INTRODUCTION

Legumes are the second most important class of crops as a source of food for humans, feed for livestock and raw materials for industry (1). Legumes possess complex secondary metabolisms and have been serving as a lynch pin of sustainable agriculture because they can acquire nitrogen efficiently through nitrogen-fixing endosymbiosis with soil bacteria called rhizobia. It is estimated that about 40–60 million tons of nitrogen are fixed annually by cultivated legumes (2), which is equivalent to about US $40 billion of fertilizer (1). Thus, understanding mechanisms that are fundamental to the legume species, especially the process of nitrogen-fixing endosymbiosis, will be of great value to healthy, low input sustainable agriculture by decreasing the use of fertilizers and improving crop yields.

We have been continuously developing the LegumeIP (3), an integrative database for comparative genomics and transcriptomics of model legumes, for the study of gene function and genome evolution in this important plant family. The original release of LegumeIP, referred to as LegumeIP 1.0 herein, hosted a total of 222,217 protein-coding genes and integrated protein family information, syntenic and phylogenetic context, tissue-specific transcriptomic profiles for three model legumes, *Medicago truncatula*, *Glycine max* and *Lotus japonicus* plus two reference plant species, *Arabidopsis thaliana* and *Populus trichocarpa*. The LegumeIP 1.0 database contained detailed gene annotations based on the UniProt (4), InterProScan (5), Gene Ontology (GO) (6) and the Kyoto Encyclopedia of Genes and Genomes (KEGG) databases (7). It provided interfaces for retrieving gene annotation, systematic synteny analysis across *M. truncatula*, *G. max*, *L. japonicas* and *A. thaliana*, as well as construction and phylogenetic analyses of gene families across the five species. The LegumeIP 1.0 database also contained a small collection of microarray gene expression data, which included 156 *M. truncatula*, 14 *G. max* and 104 *L. japonicus* array hybridization results or RNA-Seq profiles.

Emerging high-throughput technologies, such as Illumina sequencing, have generated large-scale biological data that make genomic data easily available for even more species. In the legume family, more genomes, including *M. truncatula*, *Phaseolus vulgaris* (common bean), *Cicer arietinum* (chickpea) and *Cajanus cajan* (pigeon pea) (8–12), have been completely sequenced since the publication of LegumeIP 1.0. In addition, the sequencing of the *Medicago sativa* (alfalfa), *Arachis duranensis* and *Arachis ipaensis* (the ancestors of cultivated peanut), *Vigna radiata* (mung bean) (13), *Vigna* angularis (adzuki bean) (14) and *Lupinus angustifolius* (lupin) (15) genomes are almost complete.

Meanwhile, RNA-Seq technology makes large-scale gene expression profiling possible for non-model species, where dedicated commercial microarray chips are not available. Thus, transcriptomic data are now available from various technical platforms for species such as *L. japonicas* (16), *G. max* (17), *M. truncatula* (18), *Medicago sativa* (alfalfa) (19) and *P. vulgaris* (20). These large-scale genomic and transcriptomic datasets enable and facilitate the study of fundamental mechanisms, gene functions and gene and genome evolution using comparative genomic and molecular biology approaches.

In this paper, we present the ‘*LegumeIP 2.0 - A Platform for the Study of Gene Function and Genome Evolution in Legumes*’ with a focus on the two most important new features of the database. First, we integrated genomic sequences for six legume species. These include *M. truncatula*, *G. max* and *L. japonicus* that were in the LegumeIP 1.0, three newly sequenced crop legumes, *P. vulgaris*, *C. arietinum* and *C. cajan*, and two reference plant species, *A. thaliana* and *P. trichocarpa*. The updated LegumeIP 2.0 covers 391,107 protein-coding gene sequences in total. All sequences were annotated based on sequence similarities and domain features by referring to the UniProt TrEMBL, InterProScan, GO and KEGG databases. Second, we built a brand new integrative gene expression atlas, which consists of large-scale gene expression profiles from multiple technologies for four model legumes, including 550 array hybridizations from *M. truncatula*, 962 gene expression profiles from *G. max*, 276 array hybridizations from *L. japonicas*, and 56 RNA-Seq transcriptome profiles from *C. arietinum*. All expression profiles were manually curated, annotated using free text descriptions, keywords and further hierarchically organized following the Experimental Ontology (EO) (http://bioportal.bioontology.org/ontologies/PECO?p=classes) (21,22) and Plant Ontology (PO) (http://www.plantontology.org/) (23), allowing users to browse, search and retrieve gene expression profiles for the experiments of their choice using both the EO tree view browser and comprehensive keyword searches. We believe this integrative gene atlas is essential and extremely valuable for the study of gene function and provide valuable large-scale gene expression data to biologists through user-friendly web interfaces.

## DATABASE PRODUCTION: DATA EXPANSION AND FEATURE IMPROVEMENTS

### Compilation and processing of genomic data

In LegumeIP 2.0, we have compiled and integrated gene sequences and comprehensive gene annotations for six model or crop legume genomes, including *M. truncatula*, *G. max*, *L. japonicus*, *P. vulgaris*, *C. arietinum*, *C. cajan* and two reference plant species, *A. thaliana* and *P. trichocarpa*. LegumeIP 2.0 contains 391,107 protein-coding gene sequences in total from eight plant species. Table 1 lists the included plant species and the sources of gene sequence and annotation data.

**Table 1.** A list of plant species and their gene sequence and annotation data sources in LegumeIP 2.0

Plant Species	Release Version	URL for Downloading
*Medicago truncatula*	Mt4.0	http://www.jcvi.org/medicago/display.php?pageName=General&section=Download
*Glycine max*	1.1	http://genome.jgi.doe.gov/PhytozomeV9/download/
*Lotus japonicus*	2.5	ftp://ftp.kazusa.or.jp/pub/lotus/lotus_r2.5/
*Cicer arietinum*	1.0	http://cicar.comparative-legumes.org
*Phaseolus vulgaris*	1.0	http://genome.jgi.doe.gov/pages/dynamicOrganismDownload.jsf?organism=PhytozomeV9
*Cicer arietinum*	V5.0	http://cajca.comparative-legumes.org
*Arabidopsis thaliana*	V10	https://www.arabidopsis.org/download/index-auto.jsp?dir=%2Fdownload_files%2FSequences
*Populus trichocarpa*	V3.0	http://genome.jgi.doe.gov/pages/dynamicOrganismDownload.jsf?organism=PhytozomeV9

To annotate these genes, the sequences were searched against the reference sequences from the UniProt, TrEMBL, GO and KEGG databases using BLASTP with an expect value <1e-06. Furthermore, these sequences were searched for conserved domains for inclusion of functional annotation using the InterProScan program. All transcription factors (TFs) were predicted using our previously published method (24) for improved TF annotation.

We performed systematic syntenic analysis and reconstructed cross-species gene family and phylogenic trees for the six legume genomes and the two reference plant genomes using the same methods that were used in the development of LegumeIP 1.0 (3). Supplementary Figure S1 (Supplemental Material) shows an example of three complex micro synteny views for six legume species. As we described in the LegumeIP 1.0, multiple leucine-rich repeat receptor kinases are reportedly involved in the signaling pathway that mediates early root responses to bacterial and fungi infections in epidermal tissues of root nodules (25). In LegumeIP 2.0, multiple genes (e.g., *Ca_11537*, *C.cajan_12295* and *Phvul.002G143400.1*) with the same functions could also be identified with high confidence in *C. arietinumx*, *C. cajan* and *P. vulgaris* based on micro synteny analysis across the six legume species.

### Compilation and processing of transcriptomic data

We have developed a brand new integrative gene expression atlas in LegumeIP 2.0. Because there is abundant microarray data for *M. truncatula*, *G. max* (soybean) and *L. japonicus* in public repositories, those datasets were collected, compiled and further curated for inclusion in LegumeIP 2.0. All of the expression profiles were downloaded from ArrayExpress (26), the NCBI GEO data repository (27) and other legume-specific gene expression atlases (16–18,20). The raw data were normalized by the Robust Multichip Average method (28) using the ‘affy’ module of the R (https://www.r-project.org/) software package. In addition, we included RNA-Seq-based gene expression profile data for chickpea from the NCBI SRA (29) database because there is no microarray chip designed for this organism. We adopted Bowtie (30) and RSEM (31) to map reads on the genomes, then estimated gene expressions in Fragments Per Kilobase of transcript per Million reads (FPKM) and further used edgeR (32) to normalize the final expression values. Currently, the updated gene expression atlas consists of 550 hybridizations using the Affymetrix A-AFFY-71 GeneChip for *M. truncatula*, 962 hybridizations using the Affymetrix GeneChip Soybean Genome Array A-AFFY-59, 276 hybridizations using the Affymetrix GeneChip Lotus Gene Array A-AFFY-59 and 56 Illumina RNA-Seq profiles for chickpea.

In addition to the integration of large-scale gene expression data for four legumes, one of the significant improvements in LegumeIP 2.0 is that we curated all transcriptomic datasets according to the descriptions of the original experimental designs, and further re-annotated the microarray assays (or Illumina runs) using the newly developed EO terms and PO terms. The former is a popular ontology that defines experimental conditions based on the published literature and the latter defines plant anatomical structures, which is analogous to the GO (6). Incorporating information about the experimental conditions and biological samples significantly simplifies searches of transcriptome profiling data. Furthermore, we constructed genome-scale networks for these species using our published tools (33,34).

### Database production and user-friendly web interfaces for data access

**Figure 1.**
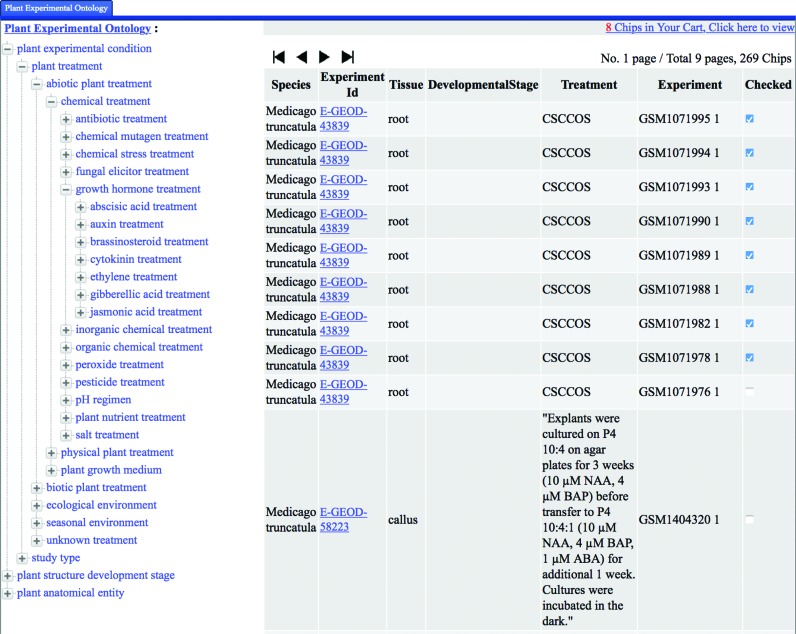
The Experimental Ontology and Plant Ontology treeview browser for selecting a subset of microarray or RNA-Seq experiments.

**Figure 2.**
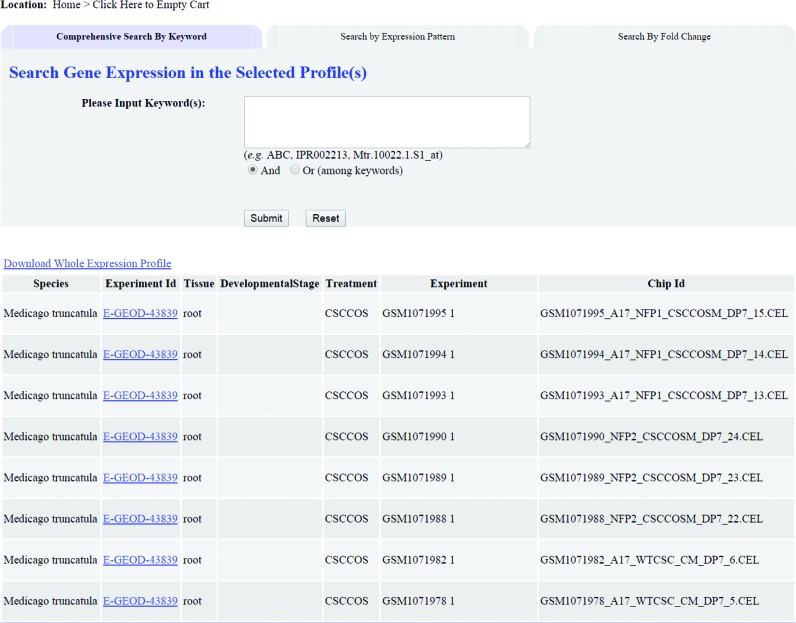
The comprehensive keyword search interface for retrieving genes’ expression in the user-selected experiments.

**Figure 3.**
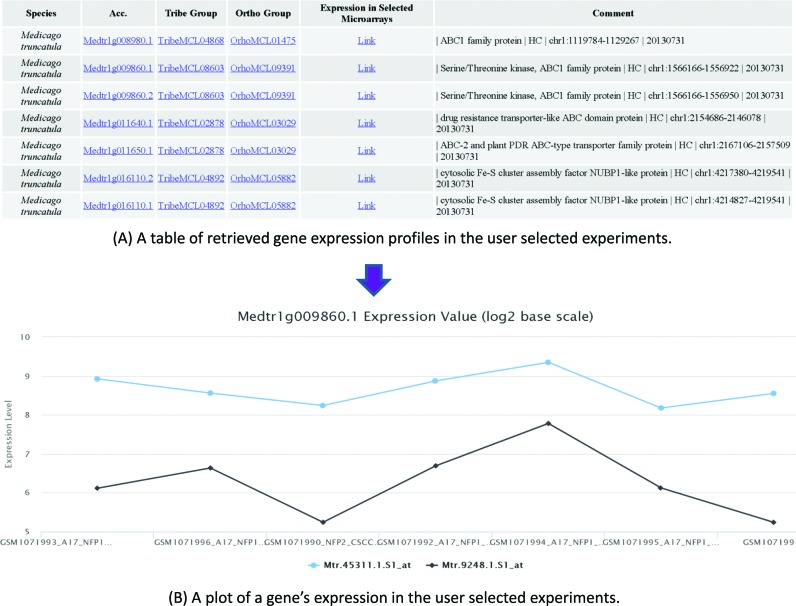
A table of retrieved gene expression profiles (**A**) and a plot of a gene's expression (**B**) in the user selected experiments.

The LegumeIP 2.0 system runs on a Linux-based Resin Java web server using MySQL as its backend data management system. Compared with the previous version, we have significantly improved the web site of LegumeIP 2.0 with more intuitive and user-friendly interfaces for searching and exploring genes, gene families and syntenic regions. For example, we developed treeview interfaces for browsing the GO-based gene annotations (Supplementary Figure S2), KEGG-based enzyme annotations, transcription factors and transporters. Furthermore, we developed user-friendly interfaces, which can be accessed under the menu ‘*Expression Atlas*’, for exploring and searching the gene expression atlas of LegumeIP 2.0, in which users can select a subset of microarray or RNA-Seq experiments and further compare expression profiles for multiple genes through EO and PO treeview browsers (Figure 1), comprehensive keyword search (Figure 2), differential expression gene analysis (Supplementary Figure S3) and advanced gene expression pattern search (Supplementary Figure S4) interfaces. Figure 3 shows a table of retrieved gene expression profiles (A) and a plot of a gene's expression (B) in the user selected experiments. We have adopted HTML 5 technologies for visualizing data in rich formatted text, tables and figures, which makes LegumeIP 2.0 compatible to all of the modern internet browsers. All of the search results can be downloaded to end-users local computers for further analysis.

## CONCLUSION AND FUTURE DEVELOPMENT

The research in legume biology has progressed significantly. Using Affymetrix GeneChip and next-generation sequencing technologies have allowed researchers to generate large-scale genomic and transcriptomic data for both model and economically important crop legumes in recent years, which necessitated an important update to our LegumeIP database. We significantly improved the database by integrating more legume species based on the latest legume genome sequencing efforts. Such improvements make LegumeIP 2.0 a more useful tool for comparative genomic study utilizing its hosted syntenic regions, cross-species gene families and phylogenetic models. In this regard, we will continuingly enhance the LegumeIP database as more legume genomes become available.

In LegumeIP 2.0, we mapped microarray and RNA-Seq experiments to the graph structures of both EO and PO. In computer science, ontology is the formal naming and definition of the types, properties and inter-relationships of the entities. Both EO and PO define terms and organize information about experiment design and plant samples in graph structures. The introduction of EO and PO can standardize the search of a transcriptomic dataset based on experimental conditions and plant samples and treatments used in the experiments, which are the main challenges in the development of large gene atlas. This development will also simplify our efforts toward continuously updating and expanding transcriptomic data in the LegumeIP system.

The LegumeIP 2.0 database features an integrative gene-atlas of four legume species and comparative genomic data, such as synteny and protein family information for all hosted legume and reference genomes. We collected raw data from the genome sequencing projects of legumes (Table 1) and transcriptomic data repositories such as the ArrayExpress (26) and the NCBI SRA (29). We performed extensive computational analysis and curation to generate these unique information which is valuable addition to other public legume databases, such as the Cool Season Food Legume Genome Database (https://www.coolseasonfoodlegume.org/), Legume Information System (35), PhaseolusGenes (http://phaseolusgenes.bioinformatics.ucdavis.edu/), SoyBase (36) and Soybean Knowledge Base (37). In future, we plan to develop application program interfaces to provide the Representational State Transfer (REST) web services to facilitate the information sharing among the legume databases.

## AVAILABILITY

LegumeIP 2.0 is free to the public and available at http://plantgrn.noble.org/LegumeIP/.
